# Natural history and clinical significance of meniscal tears over 8 years in a midlife cohort

**DOI:** 10.1186/s12891-015-0862-1

**Published:** 2016-01-05

**Authors:** Hussain Ijaz Khan, Dawn Aitken, Changhai Ding, Leigh Blizzard, Jean-Pierre Pelletier, Johanne Martel-Pelletier, Flavia Cicuttini, Graeme Jones

**Affiliations:** Musculoskeletal Unit, Menzies Institute for Medical Research, University of Tasmania, Medical Science 1 Building, Private Bag 23, 17-Liverpool Street, Hobart, TAS 7000 Australia; Department of Epidemiology and Preventive Medicine, Monash University, Melbourne, Australia; Osteoarthritis Research Unit, University of Montreal Hospital Research Centre (CRCHUM), Montreal, QC Canada

**Keywords:** Knee, Osteoarthritis, Magnetic Resonance Imaging, Radiographs, Meniscus

## Abstract

**Background:**

There is limited longitudinal data available on the natural history of meniscal tears especially in middle-aged adults with a low prevalence of osteoarthritis (OA). The aim of this study was to describe the natural history of meniscal tears over 8 years and the relationship with change in knee pain and structures.

**Methods:**

One hundred ninety eight participants [mean age 47 (28–63); 57 % female] were studied at baseline and 8 years later. Approximately half were the adult offspring of subjects who had a knee replacement performed for knee OA and the remainder were randomly selected controls. Meniscal tears/extrusion, cartilage volume/defects, bone marrow lesions (BMLs) and effusion were assessed on MRI. Knee pain was assessed using the Western Ontario and McMaster Universities Osteoarthritis Index.

**Results:**

22 % of the participants had at least one meniscal tear at any site at baseline. Over 8 years, 16 % of the participants had an increase in severity of meniscal tears while none improved. Increase in meniscal tear score was associated with worsening knee pain (β = +2.81 (+1.40, +4.22)), with offspring having a significantly greater increase in pain severity compared to controls. BMI and presence of osteophytes at baseline, but not knee injury, predicted change in tears, whereas change in meniscal tears was independently associated with cartilage volume loss, change in BMLs and change in meniscal extrusion.

**Conclusion:**

Change in meniscal tears shares risk factors with knee OA and is independently associated with worsening knee pain and structural damage suggesting that meniscal tears are on the knee OA causal pathway.

## Background

Loss of meniscal function due to tears is a potent risk factor for knee osteoarthritis (OA) and may be one of the earliest changes in the OA causal pathway [1]. Meniscal tears share common risk factors with knee OA [2, 3] and explain more of the variation in joint space narrowing (JSN) than cartilage volume [4]. Cross-sectional studies using magnetic resonance imaging (MRI) have also shown that damage to menisci in the form of tears is paralleled by other structural abnormalities such as lower cartilage volume [2] and an increased severity of cartilage defects [2] and bone marrow lesions (BMLs) [5].

Although meniscal tears are a common finding in people with asymptomatic disease [6], it is a potential source of pain associated with OA. The periphery of menisci have nociceptive innervation and it is reasonable to hypothesise that meniscal tears that extend to this area can cause pain. However longitudinal studies, conducted over 15–24 months, have shown conflicting results thus far [7, 8]. It is uncertain if change in meniscal tears is directly associated with worsening pain [7] or if both meniscal damage and pain are a result of OA through intermediate pathologies (such as BMLs and effusion) rather than a direct link between the two [8].

Furthermore, there is limited longitudinal data on the natural history of meniscal tears. It is not clear how meniscal tears change over a long period of time and how change in meniscal tears is associated with global knee structural changes. The aim of this study was to describe the natural history of meniscal tears over 8 years, the predictors of change in meniscal tears and the association between change in meniscal tears and change in knee pain and structures.

## Methods

This study was conducted as part of the Offspring study, a population-based study that began in Southern Tasmania in June 2000. Matched sampling was used to recruit the study participants (mean-age 47 (28–63) years; 57 % females). Half of the participants were the adult offspring of patients (only one parent) who had a knee replacement performed for idiopathic knee OA at any Hobart hospital from 1996 to 2000 [9]. The diagnosis was confirmed by reference to the medical records of the orthopaedic surgeons and the original radiographs when possible. The other half were age and sex matched controls, randomly selected from the population with no history of knee OA in either parent. This study includes data from the first (visit-2) and second (visit-3) follow-up visits at approximately two and ten years respectively, as we did not have the correct MRI sequence to score meniscal tears at baseline.

All procedures followed were in accordance with the ethical standards of the responsible committee on human experimentation (institutional and national) and with the Helsinki Declaration of 1975, as revised in 2000. The Southern Tasmanian Health and Medical Human Research Ethics Committee approved the protocol, and written informed consent was obtained from all participants.

Participants were excluded if they had a contraindication to MRI (including metal sutures, presence of shrapnel, iron filing in eye, or claustrophobia). Participants were also excluded if they had undergone a knee replacement surgery or did so after the commencement of the study. Knee pain and knee injury were not a basis for exclusion.

### Knee pain

Knee pain was assessed by self-administered questionnaire using the Western Ontario and McMaster Universities Osteoarthritis Index (WOMAC) at both visits [10]. Five categories of pain (walking on flat surface, going up or down stairs, at night, sitting or lying, and standing upright) were assessed separately with a 10-point scale from 0 (no pain) to 10 (most severe pain). Each category was summed to create a total pain score (range 0 to 50). Furthermore, the five categories were clinically categorized into weight-bearing pain (including walking on flat surface, going up or down stairs and standing) and non-weight-bearing pain (including pain at night and sitting or lying).

### Knee joint injury

History of knee joint injury was assessed using a self-administered questionnaire [11] which included the following questions:

“Have you ever had a previous knee injury which resulted in non-weight bearing treatment for 24 h or more?”

“If yes, then which knee?”

“Please provide further details about the injury”

### Magnetic resonance imaging

MRI of the right knee was performed as described previously [12, 13]. Knees were imaged in the sagittal plane on a 1.5-T whole-body magnetic resonance unit (Picker International, USA) using a commercial transmit-receive extremity coil. The following image sequence was used: (1) a T1-weighted fat-suppressed 3D gradient-recalled acquisition in the steady state, flip angle 55°, repetition time 58 msec, echo time 12 msec, field of view 16 cm, 60 partitions, 512 × 512–pixel matrix, slice thickness of 1.5 mm without an interslice-gap; and (2) a T2-weighted fat saturation 2D fast spin echo, flip angle 90°, repetition time 3067 ms, echo time 112 ms, field of view 16 cm, 15 partitions, 256 × 256 matrix, slice thickness of 4 mm with an interslice gap of 0.5–1.0 mm.

### Meniscal tears

Meniscal tears were assessed by a trained observer (musculoskeletal radiologist with several years of experience) on T2-weighted fat saturated (side by side) MR images at visit-2 and 3 of the study as previously described [14]. The proportion of the menisci affected by a tear was scored separately (0–2 scale; 0 = absence of a tear, 1 = simple tear of different types: longitudinal, oblique, radial or horizontal, 2 = macerated tear signifying loss > 50 % area of meniscal tissue) at the anterior, middle, and posterior horns. Anterior, middle and posterior scores were summed to create medial and lateral meniscal tear scores. The intra- and inter-observer correlation coefficient (expressed as intraclass correlation coefficient (ICC)) ranged from 0.86 to 0.96 [15].

### Meniscal extrusion

The extent of meniscal extrusion on the medial or lateral edges of the tibial femoral joint space, not including the osteophytes, was evaluated at visit-2 and 3 for the anterior, body, and posterior horns of the menisci on T1-weighted gradient echo MR images, as previously described [15]. A score from 0 to 2 was used (0 = no extrusion, 1 = partial meniscal extrusion, and 2 = complete meniscal extrusion with no contact with the joint space). The scores of anterior, body and posterior horns of medial or lateral menisci were summed to create a total meniscal extrusion score for each of the medial and lateral tibiofemoral compartments which had a possible range from 0 to 6. The intra- and inter-observer correlation coefficient ranged from 0.85 to 0.92 for meniscal extrusion [14].

All knees were evaluated for the presence of meniscal extrusion regardless of whether they had a meniscal tear or not.

### Cartilage volume

Tibial and femoral cartilage volume was assessed on T1-weighted gradient echo MR images using Osiris (University of Geneva, Switzerland) and Cartiscope (ArthroLab, Montreal, Canada) software respectively at visit-2 and 3, as previously described [12, 15]. The coefficient of variation (CV) for intra-observer repeatability ranged from 2.0–2.2 % for both tibial and femoral cartilage volume measurements [16, 17]. Total cartilage volume was calculated as: tibial + femoral cartilage volume.

### Cartilage defects

Cartilage defects were assessed on T1-weighted gradient echo MR images on a 0–4 scale (0 = normal; 1 = focal blistering/signal changes; 2 = <50 % thickness loss; 3= > 50 % thickness loss; 4 = full thickness defect) at visit-2 and 3, as previously described [18]. Intraobserver reliability ranged from ICC of 0.89–0.90 [18]. Interobserver reliability was assessed in 50 MR images and yielded an ICC of 0.85–0.90 [18].

### Bone marrow lesions

BMLs were assessed on T2-weighted fat saturated MR images at visit-2 and 3 and were defined as areas of increased signal adjacent to the subchondral bone [10]. One trained observer scored the BMLs by measuring the maximum area of the lesion in a specific compartment. The observer manually selected the MRI slice with the greatest BML size. The BML with the highest score was used if more than one lesion was present at the same site. The ICC for intra-observer reliability, assessed on 40 MR images, was 0.97.

### Effusion

Effusion was assessed in the supra-patellar pouch on T2-weighted fat saturated MR images at visit-2 and 3 on a 0–3 scale [19]. Grade-0 signified absence of fluid over the upper margin of the patella in a sagittal image; Grade-1 signified some fluid above the upper margin of the patella but the length of the fluid column shorter than that of the patella; Grade-2 signified a fluid column above the upper margin of patella longer than the length of the patella; Grade-3 signified a fluid column above the upper margin of patella longer than the length of the patella with a thickness of ≥ 1 cm. Intra-observer reliability was assessed in 50 MR images and yielded an ICC of 0.89–0.98. Pathological effusion was defined as any effusion score ≥2.

### Radiography

A standing anteroposterior semiflexed view of the right knee (at 15° flexion) was performed in all participants at baseline and 10 years. Radiographs were scored individually for osteophytes and joint space narrowing, as described previously [16]. Each of the following four features was scored on a scale from 0 to 3 (0 = normal and 3 = severe): medial joint space narrowing (JSN), lateral JSN, medial osteophytes (femoral and tibial combined) and lateral osteophytes (femoral and tibial combined). Each score was arrived at by consensus with two readers simultaneously assessing the radiograph with immediate reference to the Osteoarthritis Research Society International (OARSI) atlas [20]. A non-zero score in either joint space narrowing or osteophytosis was regarded as evidence of radiographic osteoarthritis (ROA). Reproducibility was assessed in 50 radiographs, two weeks apart, and yielded an ICC of 0.99 for osteophytes and 0.98 for JSN.

Readers for all the scans were either musculoskeletal radiologists with several years of experience in OA research or health professionals trained by musculoskeletal radiologists. Readers were not blinded to the chronological sequence of the radiographs and MRI scans.

#### Statistical analysis

Change in all MRI structures and leg strength was calculated as: Visit-3 score – Visit-2 score.

*T*-test and Chi-square tests were used to describe the baseline characteristics of the participants with or without any change in mean meniscal tear score. *T*-test was further used to compare change in meniscal score between offspring and control groups. Poisson regression analysis was used to examine the predictors of change in meniscal tears and the association between change in meniscal tears and change in meniscal extrusion. Linear regression analysis was used to describe the association between change in meniscal tears and change in pain, cartilage volume loss and change in BMLs. Multivariable analyses were adjusted for demographics, body mass index (BMI), offspring-control status and knee structures (global knee structural factors known to be associated with the presence of meniscal tears or knee pain). Further analysis was performed to explore any offspring-control interaction in the multivariable models for all the above mentioned associations.

A P-value of less than 0.05 (two-tailed) was considered statistically significant. All statistical analyses were performed on Intercooled Stata 12.0 for windows (StataCorp LP).

## Results

A total of 198 subjects (57 % female, mean age 47 years) had complete MRI measures at visit-2 and 3. There were no significant differences in baseline characteristics between those lost to follow-up (*n* = 133) and the participants in our study in terms of age, sex, BMI and ROA (data not shown).

### Natural History

**Fig. 1:**
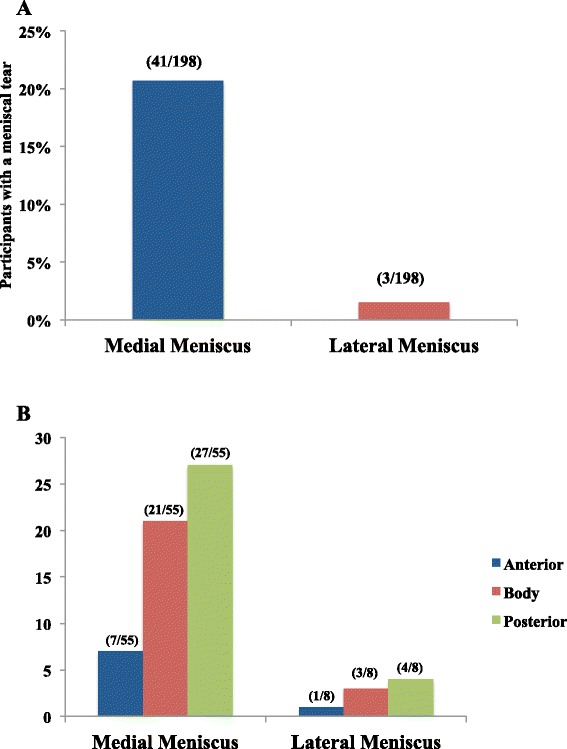
Prevalence and natural history of meniscal tears. **a** Prevalence of meniscal tears at visit 2; **b** Site-specific distribution of meniscal tears at visit 2

Figure 1a describes the prevalence of meniscal tears at visit-2. 22 % of the participants (44/198) had at least one meniscal tear at any site. 41/44 participants had at least one meniscal tear at any of the three meniscal sites (anterior, body or posterior) in the medial compartment, whereas only 3 participants had at least one meniscal tear in the lateral compartment. None of the participants had a meniscal tear in both compartments.

Forty one participants with medial meniscal tears had 55 meniscal tears in total at all sites. 29/41 participants had a single meniscal tear at any site (anterior, body or posterior), 10/41 participants had a meniscal tear at 2 sites and 2/41 participants had a meniscal tear at all 3 sites. Medial posterior was the most commonly affected site (27/55), followed by medial body (21/55) and medial anterior sites (7/55) (Fig. 1b). 37/55 meniscal tears were simple tears, whereas 18/55 were macerated tears.

Three participants with lateral meniscal tears had 8 meniscal tears in total at all sites. 1/3 participant had a meniscal tear at 2 sites and 2/3 participants had meniscal tears at all 3 sites. Lateral posterior was the most commonly affected site (4/8), followed by lateral body (3/8) and lateral anterior sites (1/8) (Fig. 1b). 5/8 meniscal tears were simple tears, whereas 3/8 were macerated tears.

The majority of participant’s menisci (84 %) remained stable over 8 years. 16 % of the participants (31/198) showed an increase in mean meniscal score—including incident tears (14/31) and increase in the severity of existing tears (17/31). Most of these changes affected the medial meniscus (87 % (27/31)).

Most of the participants showed an increase at the posterior meniscal site (15/31), followed by body (12/31) and anterior (4/31) sites. None of the participants with a meniscal tear at visit-2 showed an improvement in meniscal tear score over 8 years.

Table 1 describes the (visit-2) characteristics of participants with and without any increase in mean meniscal tear score over 8 years. Participants with any increase in mean meniscal score were significantly older, heavier, had a higher percentage of offspring, prevalence of ROA, total femoral cartilage volume, total mean cartilage defect score, tibial bone area and prevalence of supra-patellar effusion compared to participants without any increase in mean meniscal score. Participants with any increase in mean meniscal tear score also had a higher percentage of male participants, worse pain score and a higher prevalence of BMLs but these differences did not reach statistical significance.

**Table 1:** Characteristics (at visit-2) of participants with and without any change (incident tears and increase in score) in tears over 8 years

	Any change (n = 31)	No change (n = 167)	*p*-value
Age (years)	**50.06 ± 6.35**	**47.37 ± 6.49**	**0.046**
Male (%)^a^	57	39	0.069
BMI (kg/m^2^)	**29.51 ± 7.10**	**26.77 ± 4.38**	**0.008**
Offspring (%)^a^	**72**	**45**	**0.008**
Any ROA (%)^a, b^	**33**	**18**	**0.046**
WOMAC pain (mean)	4.77 ± 7.14	2.63 ± 4.71	0.051
Total tibial cartilage vol (mm^3^)	4868.43 ± 1012.85	4500.62 ± 1062.66	0.093
Total femoral cartilage vol (mm^3^)	**9562.64 ± 2377.24**	**8531.51 ± 2269.82**	**0.047**
Total cartilage defects (mean)	**5.24 ± 2.04**	**3.80 ± 1.43**	**<0.001**
Total tibial bone area (mm^2^)	**3273.17 ± 473.89**	**3079.38 ± 473.01**	**0.049**
Any bone marrow lesion (%)^a^	59	50	0.380
Any pathological effusion (%)^a^	**55**	**34**	**0.028**

Mean ± standard deviation except for percentages

Bold font denotes statistically significant (*p* = <0.05) results

^a^Determined by Chi square test, others by *t*-test

^b^Assessed at the baseline visit; the rest assessed at visit-2

The majority of meniscal tear change occurred in the offspring group and this was significant at the total medial, total posterior and the total knee sites in comparison to the control group (all p < 0.05).

### Predictors of change

Table 2 describes predictors of change in total knee meniscal tears over 8 years. Age at visit-2, BMI, history of knee injury, cartilage defects, BMLs, JSN and osteophytes significantly predicted change in meniscal tears in unadjusted analysis. Only BMI and osteophytes independently predicted change in meniscal tears in the fully adjusted model. BMI showed a significant association in all compartments including anterior, body and posterior meniscal sub-groups whereas osteophytes predicted change in only total anterior and posterior tears (data not shown).

**Table 2:** Predictors of change in total knee meniscal tears over 8 years

	Change in total knee meniscal tears over 8 years	
	Unadjusted	Adjusted^a^
	Risk ratio (95 % CI)	Risk ratio (95 % CI)
	**1.06**	1.05
Age	**(1.02, 1.11)**	(0.98, 1.21)
	**1.09**	**1.11**
BMI	**(1.03, 1.15)**	**(1.04, 1.17)**
	**2.16**	1.91
Knee Injury	**(1.08, 6.01)**	(0.93, 3.92)
	**1.26**	0.77
Cartilage defects	**(1.05, 1.52)**	(0.54, 1.09)
	**1.57**	0.87
BMLs	**(1.06, 2.32)**	(0.33, 2.29)
	**3.17**	2.11
JSN	**(1.41, 7.16)**	(0.74, 6.03)
	**1.79**	**1.78**
Osteophytes	**(1.29, 2.47)**	**(1.17, 2.71)**

Bold font denotes statistically significant (*p* = <0.05) results

(No significant offspring-control interaction for any of the above mentioned associations)

^a^adjusted for age/BMI/knee injury, offspring-control status, cartilage defects at visit-2, BMLs at visit-2 and/or ROA at visit-1

### Pain

30/44 participants who had a meniscal tear reported knee pain at baseline.

Table 3 describes the association between change in meniscal tears and change in pain over 8 years. Increases in total knee meniscal tears was independently associated with increases in total knee pain, pain on each individual WOMAC sub-scale and in weight bearing and non-weight bearing pain over 8 years in the whole population. There was also a significant offspring-control interaction at all sites with offspring showing significantly greater increases in pain per unit increase in meniscal tears compared to controls.

**Table 3:** Association between change in meniscal tears and change in pain over 8 years

	**Change in pain over 8 years**	
Change in total knee	Unadjusted	Adjusted^a^
meniscal tears	β (95 % CI)	β (95 % CI)
Whole group	**+2.87 (+1.84, +3.90)**	**+2.81 (+1.40, +4.22)**
–Offspring	**+3.73 (+2.56, +4.89)**	**+2.84 (+1.22,+4.46)**
–Controls	−0.48 (−2.72, +1.75)	−0.92 (−4.20, +2.36)
	**Change in pain subscales over 8 years**	
	Change in pain while lying in bed	
Whole group	**+0.89 (+0.64, +1.14)**	**+0.82 (+0.46, +1.18)**
	Change in pain while sitting	
Whole group	**+0.45 (+0.22, +0.67)**	**+0.35 (+0.04, +0.67)**
	Change in pain while standing	
Whole group	**+0.55 (+0.31, +0.80)**	**+0.62 (+0.31, +0.94)**
	Change in pain while walking on flat surface	
Whole group	**+0.56 (+0.35, +0.77)**	**+0.49 (+0.20, +0.78)**
	Change in pain while climbing stairs	
Whole group	**+0.33 (+0.02, +0.65)**	**+0.59 (+0.15, +1.02)**
	Change in pain in non-weight bearing	
Whole group	**+1.34 (+0.90, +1.78)**	**+1.18 (+0.56, +1.80)**
	Change in pain in weight bearing	
Whole group	**+1.49 (+0.82, +2.16)**	**+1.66 (+0.75, +2.58)**

Bold font denotes statistically significant (*p* = <0.05) results

(Note: Significant offspring-control interaction at all sites and sub-scales for the association between change in meniscal tears and change in pain)

^a^Adjusted for age, sex, BMI, offspring-control status, change in BMLs, change in cartilage defects, change in meniscal extrusion, change in effusion, history of knee injury and ROA at visit-1

### Structural changes

Table 4 describes the association between change in meniscal tears and knee structures on MRI over 8 years. Change in meniscal tears was independently associated with cartilage volume loss in the medial compartment only, increases in medial, lateral and total tibiofemoral BML area and with a higher risk of change in medial meniscal extrusion.

**Table 4:** Association between change in meniscal tears and knee structures on MRI over 8 years

	β (95 % CI)	β (95 % CI)	Risk ratio (95 % CI)
	Adjusted^a^	Adjusted^a^	Adjusted^a^
Change in tears (site)	Cartilage volume loss	Change in BMLs	Change in meniscal extrusion
	Total tibiofemoral	Total tibiofemoral	Total knee
Total knee	−52 (−208, +102)	**+0.41 (+0.29, +0.52)**	N/A
	Medial tibiofemoral	Medial tibiofemoral	Medial meniscus
Total medial	**−176 (−302, −49)**	**+0.33 (+0.22, +0.43)**	**1.53 (1.14, 2.03)**
	Lateral tibiofemoral	Lateral tibiofemoral	Lateral meniscus
Total lateral	+143 (−731, +1018)	**+0.26 (+0.10, +0.41)**	N/A

Bold font denotes statistically significant (*p* = <0.05) results

(No significant offspring-control interaction at any site for the association between change in meniscal tears and change in BMLs)

Note: Not enough change in lateral meniscal extrusion for analysis due to lack of power

^a^adjusted for age, sex, bmi, offspring-control status, cartilage volume loss, change in BMLs, cartilage defects and meniscal extrusion, and ROA at visit-1

There was no significant association between change in meniscal tears and change in cartilage defects at any site in the fully adjusted model.

Only two participants underwent knee surgery between baseline and visit-3 and on both occasions the surgery was not a menisectomy or a joint replacement. Further adjustment for knee surgery did not change the effect size considerably for any of the associations described earlier (data not shown).

## Discussion

This study documents the natural history of meniscal tears over 8 years. In this midlife cohort meniscal tears were common with 22 % of the participants suffering from at least one. 16 % of the participants showed an increase in severity and none improved over 8 years. BMI and osteophytes independently predicted an increase in meniscal tears over 8 years. Change in meniscal tears was independently associated with an increase in knee pain severity, with offspring showing a greater increase in the severity of pain per unit change in meniscal tears compared to the control group. Change in meniscal tears was independently associated with cartilage volume loss, change in BMLs and meniscal extrusion over 8 years.

Majority of the meniscal tears (55/63) at visit-2 affected the medial meniscus. Medial posterior site showed the highest prevalence followed my medial body sites. Previous studies by Englund et al. [6] in older adults and by K. A Beattie et al. [21] in middle-aged adults showed a similar distribution in cross-sectional studies as well. Although the majority of the menisci remained stable over the course of 8 years, 16 % showed an increase in severity over time. Again medial posterior was the most commonly affected site for both incident meniscal tears and worsening meniscal tear grades. Of note, none of the meniscal tears improved over the course of the study, unlike other knee structures such as BMLs [22] and cartilage defects [13] as previously shown in this cohort. Previously Dillon et al. [23] followed 22 patients with 27 intra-meniscal lesions with signal intensity changes on MRI but no tears on arthroscopy. After 27 months only 2 completely disappeared. Similarly Boegard et al. [24], followed 47 patients and found that only 2 meniscal tears out of 54 improved and none disappeared over 2 years. Meniscal tears, unlike other knee structures, do not seem to have the capacity to regenerate or improve over time. Slight discrepancies in the above mentioned studies could be due different populations, a longer follow-up period resulting in less measurement error in the present study and a possibly a more severe disease process in the offspring sub-group.

High BMI was the most consistent independent risk factor for increase in meniscal tear severity. A previous cross-sectional study from the present cohort showed that a higher BMI is positively associated with prevalent meniscal tears [2]. Our findings are consistent with Baker et al. [25] but differ from Englund et al. [3], who found a significant association between BMI and meniscal extrusion but not tears. A recent meta-analysis examining risk factors for meniscal tears concluded that a high BMI is a moderate risk factor for developing meniscal tears along with occupational and recreational joint loading [26]. Osteophytes at visit-1 also predicted worsening of meniscal tears. Osteophytes are thought to be an early instigating factor in the OA causal pathway and their true prevalence is under estimated on radiographs [27]. Beattie et al. [21] showed, using peripheral MRI, that many peripheral osteophytes are missed by standard radiographs and their presence corresponds with degenerative meniscal changes at the same site. Presence of osteophytes in our study also showed a significant association with change in meniscal tears at the peripheries (anterior and posterior) and not at the meniscal body site. Interestingly, history of knee injury was not independently associated with meniscal tear increase. Previously, Englund et al. [3] have shown that history of knee injury is a strong risk factor for developing meniscal tears but they did not adjust for potential confounders. Similarly, we found a significant association between knee injury and meniscal tears in unadjusted analysis but this association did not persist in the fully adjusted model. These findings suggest that the changes in meniscal tears are not due to mechanical factors only and are mainly a part of an active osteoarthritic process.

Previously in this cohort, we showed a cross-sectional association between presence of meniscal tears and increased pain [2]. In a longitudinal study, Zanetti et al. [7] found that asymptomatic participants with a meniscal tear are more likely to develop knee pain than participants without one. Englund et al. [8] on the other hand concluded that any association between meniscal damage and knee pain seems to be present because both pain and meniscal damage are related to OA and not because of a direct link between the two. Our study is the first study to show an independent longitudinal association between increasing severity of meniscal tears and worsening pain, including pain on all individual WOMAC sub-scales, as well as both weight bearing and non-weight bearing pain. Previous studies have also suggested that meniscal tears appear to cause symptoms only when macerated tears extrude and damage collateral ligaments or when bone marrow abnormalities are present [28]. Results in this study were independent of change in meniscal extrusion and BMLs as well as localised inflammation as assessed by knee effusion, suggesting meniscal tears may be one of the most important knee structures in relation to pain.

Every unit increase in meniscal tears in the offspring group resulted in a greater increase in pain compared to the controls. Previously in this cohort, we found similar differences between the two groups when looking at the association between change in BMLs and pain [22]. A possible explanation could be the differences in the pain perception pathways of the two groups. Of note, polymorphisms in COMT and TRPV1 genes have recently been identified which could alter the processing of nociceptive pain associated with OA [29]. Another possible explanation could be that meniscal pathology in the offspring is morphologically different but this could not be differentiated on MRI.

Biomechanical studies have shown that the function of the meniscus is to reduce contact stress by enlarging the contact surface and shock absorption [30]. Meniscal function can be either lost due to meniscal tears or meniscal extrusion. Meniscal tears, especially macerated tears, are a possible risk factor for meniscal extrusion [31] and findings from this study confirm this. Loss of meniscal function can potentially damage articular cartilage and sub-chondral bone. Cross-sectional studies have shown that prevalent meniscal tears are associated with decreased cartilage volume [2] and BMLs [32]. Chang et al. [33] showed that meniscal tears are longitudinally associated with site specific cartilage loss. Findings in this study are in agreement with the latter study, as we found that meniscal tear increases were associated with medial cartilage loss independent of other knee structural changes. The present study is also the first to show a longitudinal association between increase in meniscal damage and increase in BML size. Menisci aid in load distribution and BMLs have been shown to be a consequence of abnormal loading within the knee joint [34], which explains the association between the increasing severity of these structural abnormalities. High BMI and osteophytes are possibly the early instigating factors that predict increasing severity of meniscal tears and then change in meniscal tears is associated with other structural changes such as meniscal extrusion, cartilage volume loss and BMLs.

A strength of our study is that it has the longest follow-up period of any OA cohort using MRI. A limitation of our study is a significant loss to follow up. Loss to follow-up can be a potential source of bias, however re-analysis of the data using inverse probability weighting did not change any of the results, indicating robust results. This cohort also has a wide age range (28–63 years old) as the inclusion criteria did not specify any specific age range. However all the results described in this study were adjusted for age. Another limitation was the absence of radiographs at visit-2 of the study, as we did not anticipate any major changes on radiographs due to young mean-age of the cohort with a low osteoarthritis disease burden and a short follow-up period of 2 years. Furthermore, we did not analyse different types of simple tears (longitudinal, oblique, radial or horizontal) separately due to a low number of individual lesions and hence insufficient power for analysis.

## Conclusion

Change in meniscal tears shares common risk factors with knee OA and is independently associated with worsening knee pain and structural damage suggesting that meniscal tears are on the knee OA causal pathway.
